# Smart stimuli-responsive injectable gels and hydrogels for drug delivery and tissue engineering applications: A review

**DOI:** 10.3389/fbioe.2023.1104126

**Published:** 2023-02-22

**Authors:** Saba Salehi, Seyed Morteza Naghib, Hamid Reza Garshasbi, Sadegh Ghorbanzadeh, Wei Zhang

**Affiliations:** ^1^ Nanotechnology Department, School of Advanced Technologies, Iran University of Science and Technology and Biomaterials and Tissue Engineering Department, Breast Cancer Research Center, Motamed Cancer Institute, Iran University of Science and Technology (IUST), ACECR, Tehran, Iran; ^2^ State Key Laboratory of Structure Analysis for Industrial Equipment, Department of Engineering Mechanics, Dalian University of Technology, Dalian, China

**Keywords:** injectable, stimuli-responsive, hydrogel, drug delivery, tissue engineering

## Abstract

Hydrogels are widely used biomaterials in the delivery of therapeutic agents, including drugs, genes, proteins, etc., as well as tissue engineering, due to obvious properties such as biocompatibility and their similarity to natural body tissues. Some of these substances have the feature of injectability, which means that the substance is injected into the desired place in the solution state and then turns into the gel, which makes it possible to administer them from a way with a minimal amount of invasion and eliminate the need for surgery to implant pre-formed materials. Gelation can be caused by a stimulus and/or spontaneously. Suppose this induces due to the effect of one or many stimuli. In that case, the material in question is called stimuli-responsive because it responds to the surrounding conditions. In this context, we introduce the different stimuli that cause gelation and investigate the different mechanisms of the transformation of the solution into the gel in them. Also, we study special structures, such as nano gels or nanocomposite gels.

## Introduction

Gels are polymer networks that are expanded by a fluid that occupies their entire volume (Veloso et al., 2021; Wang et al., 2022). Different gels with different fluid components are used in medical applications (Mohammad et al., 2021). Hydrogels with water fluid components are the most widely used type of materials, but there are other types, such as organogels, some members of this family that have low toxicity can be used in biological applications (Wang Y. et al., 2021). In other words, hydrogels are hydrophilic polymer networks that may absorb water up to thousands of times their dry weight (Ghazinezhad et al., 2022). The abundant use of hydrogels in medical applications is due to the extraordinary properties that these materials show (Zainal et al., 2021). For example, hydrogels are usually biocompatible. This characteristic is due to their high water content and similarity to the body’s natural tissue in terms of chemical composition and mechanical properties (Pang et al., 2020). In addition, the target site may have damaging conditions for the cargo (Sabourian et al., 2020). Hydrogels can protect cells and sensitive drugs such as peptides, proteins, oligonucleotides, and DNA inside them, preventing their association with enzymes and ROS (Gačanin et al., 2020).

Now, we will explain each application and the advantage of using hydrogels. First, we examine the application of tissue engineering. Tissue engineering uses biomaterials and, in some cases, cells and growth factors to improve the function, replace, restore or repair a lost or damaged body tissue. In tissue engineering, hydrogels are either made from the beginning with sufficiently large pores. Cells are placed in them, or the pores may be created by dissolving or destroying the hydrogel or releasing growth factors. The cells penetrate these places and, after growing and proliferating, secrete their extracellular matrix (ECM) and regenerate the tissue in this way.

Hydrogels work well in delivering nutrients and GF to cells and removing the products of cellular processes from them. Also, cell adhesive ligands can be easily attached to them. On the other hand, highly hydrated hydrogels mimic the physical and chemical environment of ECM; especially, injectable hydrogels have a microstructure similar to ECM, and for this reason, a good physical integration between these two is created. Also, hydrogels support cell proliferation due to their porous network.

The second application that is examined in this article is drug delivery. The advantage of using hydrogels in drug delivery mostly refers to regulating the processes’ kinetics. For example, the network porosity can be easily adjusted to control the speed of drug penetration into the surrounding tissues. In addition, it is possible to deliver both hydrophilic and hydrophobic drugs with these materials.

Among the various gels, some formulations can be injected into the body in a sol state and turned into a gel after applying the stimulating effect *in situ*. These materials were highly regarded for the following reasons. (Huang et al., 2019; Ayoubi-Joshaghani et al., 2020; Bertsch et al., 2022).

• Cells and bioactive molecules can easily and, through a simple mixing process, be homogenously combined with the hydrogel precursor or the same solution, which also solves cell adhesion problems (Ishihara et al., 2020; Rajabi et al., 2021; Xue et al., 2021).
• In the application of tissue engineering, injecting biomaterials instead of invasive surgeries imposes less pain on the patient (Deus et al., 2020; Zeimaran et al., 2021).
• In addition, tissue recovery is faster and ultimately leaves a smaller scar. In addition, the injection of these materials into the defect area and its gelation makes the geometry of the implant perfectly match the defect and eliminate the need for pre-formed patient-specific scaffolds.
• On the other hand, in drug delivery, injection and local delivery of carriers reduce systemic toxicity (de Araújo et al., 2019; Sun et al., 2020).

Gelation of solutions occurs by creating cross-links between monomers, which this links can be chemical or physical (Marco-Dufort and Tibbitt, 2019; Peppas and Mikos, 2019). Chemical cross-links are created due to chemical reactions and are covalent bonds (Cao L. et al., 2019). These bonds are stronger than physical bonds and, as a result, are mechanically more stable, so these types of gels by chemical bonds are not diluted in environmental fluids and do not penetrate or move away from the intended site. In addition, they are physically compatible with more types of tissues and create a good and long-term connection with the surrounding tissue (Shen et al., 2023). These connections are permanent, and reversibility does not occur in them. In the past, these gels were mostly used for pre-formed implants, but with new techniques, it became possible to inject them.

Another type of cross-link is physical and non-covalent; chemical reactions do not play a role in their formation (Chen et al., 2019). The most important interactions that lead to gel production in this category are hydrogen bonding, dipolar-dipolar, and hydrophobic interactions (Anderson et al., 2020; Zhou et al., 2020; Yang et al., 2022). These relatively weak interactions have reversible nature, which means that the substance can turn into a gel in the environment outside the body, then during injection into the body, due to the shear stresses of the injection, the bonds are broken and turn into a sol, and after a while, it returns to the gel state again (Liu et al., 2020; Bai et al., 2022; Zou et al., 2022).

Nanogels and nanocomposite gels are two important examples of gels, which will be explained below. Nanogels are gels with a size of 1 to generally 200 nm, so in addition to the properties of hydrogels, due to their specific dimensions, they have characteristics that we will review below (Liu X. et al., 2022). These substances can escape the elimination process by the body’s immune system. Also, due to the network structure after injection, they have a high circulation time in the bloodstream until they reach the target location, which increases the probability of targeting (Taghipour et al., 2022). In addition, the transfer of a hydrophilic surface to the nanogel increases the circulation time in the bloodstream and prevents particle absorption by immune mononuclear phagocytic cells (Rabiee et al., 2019). Due to their high surface-to-volume ratio, they have a large surface area for biological conjugation and cargo loading (Rabiee et al., 2019). The last item can be carried out without chemical reaction, effectively maintaining drug activity in drug delivery applications. Due to their small dimensions, they have a high response speed to the stimulus. Also, due to their small size and soft structure, they can reach small capillaries and penetrate the target tissues through transcellular and paracellular routes. Easy synthesis, the ability to adjust the size, stability in the presence of serum, the possibility of controlled release, and the ability to deliver hydrophobic drugs and sensitive compounds of protein or nucleic acids are some of the advantages of this class of materials (Amina and Guo, 2020; Curreri et al., 2021).

In addition, they can be used for targeted drug delivery, for example, to tumors. In this way, in addition to passive targeting, i.e., the inherent tendency of nanogels to accumulate in tumors, nanogels can perform active targeting by modifying the surface, for example, with a ligand. These substances can be injected intravenously, and after accumulating at the tumor site, they can be cross-linked by a stimulant and turned into a bulk gel (Cao et al., 2020). These substances overcome the challenges of conventional drug treatments, including low bioavailability. They reduce previous treatments’ adverse reactions and side effects (Dalir Abdolahinia et al., 2022). However, despite the advances, the performance of nanocarriers usually has side effects. These structures generally have minimal toxicity.

Nevertheless, most synthetic nanogels have the risk of toxic compounds, so some of them have been prevented from using (Ma et al., 2022). Nanocomposite hydrogels are hydrogels in which nanomaterials are incorporated. Nanostructures can also be created *in situ* in the hydrogel matrix (Kuang et al., 2019). When nanoparticles can cause unwanted biological responses in the body (e.g., immune response), the hydrogel can prevent the two from directly interacting. In smart nanocomposite gels, the type of nanoparticle determines the type of stimulus (es) and enhances delivery in different body parts (Wen et al., 2020).

Reinforcing nanostructures form a strong link with many matrixes polymer chains because they have a high surface-to-volume ratio. This results in the composite having a high strength and toughness, which is crucial in the application of tissue engineering. The reinforcement can also give a kind of biological activity, such as angiogenesis or osteogenesis induction, to the hydrogel. In addition, it can improve the gelation time and biodegradation. In some systems, surface-modified nanoparticles can act as gelators and cause lateral chemical cross-linking of the network (Mellati et al., 2021). Conventional hydrogels are usually used to carry hydrophilic drugs and release hydrophobic drugs quickly, but nanocomposites can carry hydrophobic drugs. In addition, nanocomposites have a higher swell/deswell rate and mimic natural tissues more (Kurian et al., 2022).

## Endogenous stimuli

Internal stimuli are the conditions applied by the environment inside the body after injection to the gel and inducing gelation. All the gels mentioned in this category are physical.

### Temperature

**FIGURE 1:**
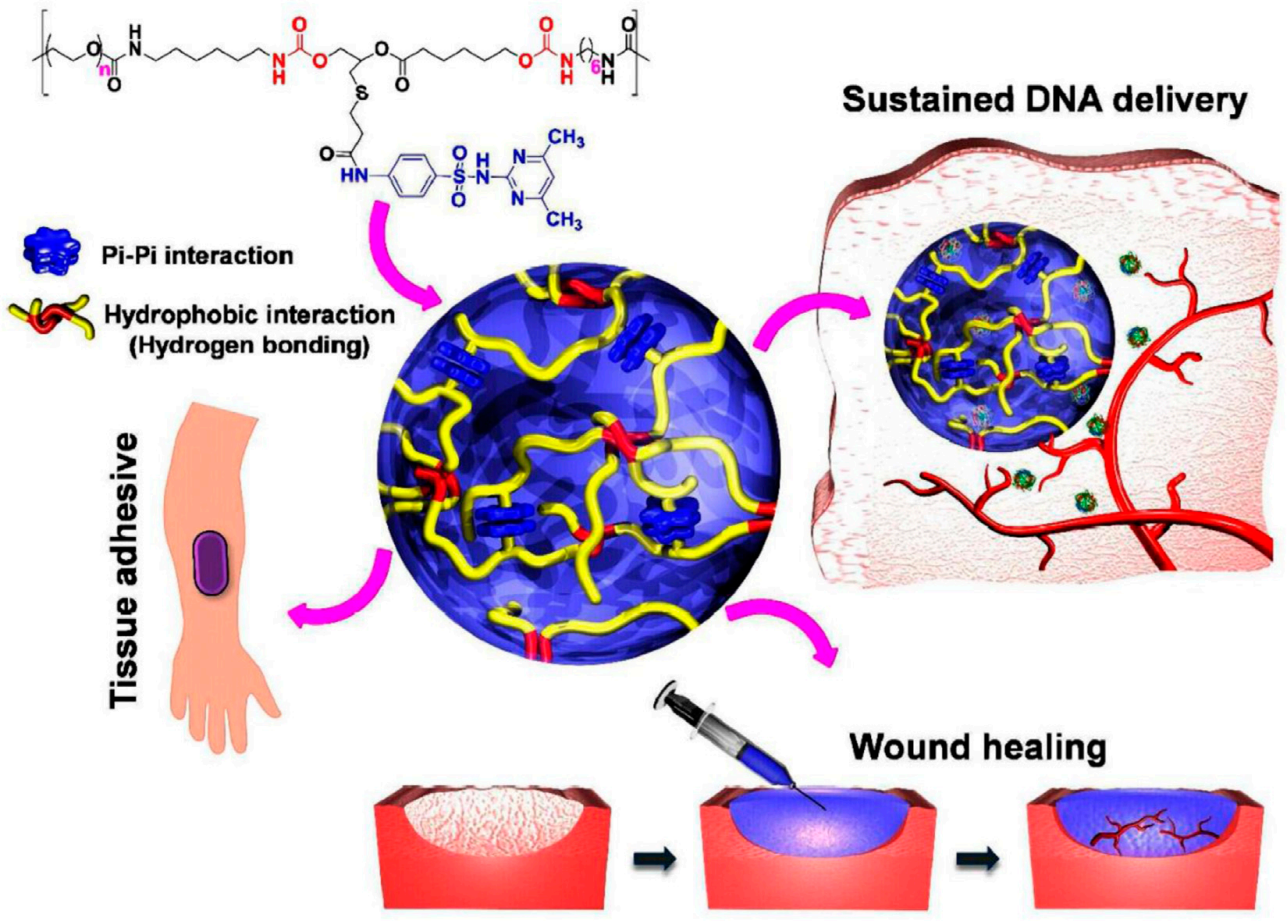
The idea of PEG-PSMEU copolymers’ sol-to-gel phase transition and their numerous biomedical applications. i) Following subcutaneous delivery of PEG-PSMEU copolymer sols containing polyplex, a viscoelastic gel was created that controlled the release of the polyplex. ii) Similar to adhesives inspired by mussels, the PEG-PSMEU copolymer hydrogel easily penetrates onto the skin’s surface. iii) The hydrogels’ tissue adhesion properties are used in wound healing to close wounds and restore tissue structure and function. Reprinted with permission from (Le et al., 2018). Copyright 2018 American Chemical Society.

**FIGURE 2:**
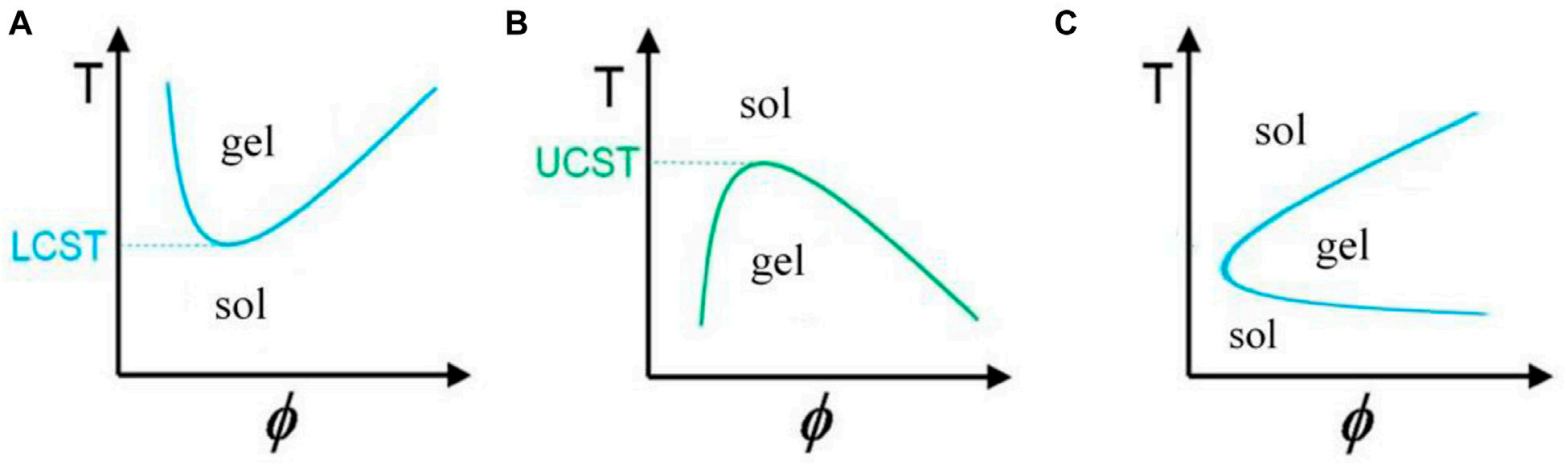
Graph of temperature changes with volume fraction of polymer in solution for gels with **(A)** negative, **(B)** positive (Ward and Georgiou, 2011), and **(C)** reversible temperature behavior.

The temperature at which gelation occurs depends on the concentration of monomers in the fluid. For each substance in a specific concentration, gelation occurs at the lowest temperature, called the lower critical solution temperature or (LCST); this temperature point is called the cloud point (Figure 2A). These gels have a negative temperature dependence, which means that they shrink with increasing temperature, and for example, if they contain drugs, the release of the drug from them is limited. These gels are widely used because they can be dissolved at room temperature (25°C) and turned into a gel by injecting them into the body and reaching the temperature of the material to about 37°C (Nguyen et al., 2019). The gelation mechanism in this category of materials is that the hydrogen bond between the side groups of monomers and water in the solution weakens with increasing temperature, and gelation and contraction occur with the strength of hydrophobic interactions of the main chain, and the dehydrated polymer is formed (Figure 1) (Le et al., 2018). PNIPAM Poly (N-isopropyl acrylamide) is the most famous member of this family with an LCST of about 32°C, and its shrinking behavior occurs with a state transition from the extended helix to the coiled-coil. It is possible to increase the gelation temperature by copolymerizing this polymer with hydrophilic monomers and decreasing this temperature with hydrophobic monomers. The first case is used because the gelation temperature close to body temperature are desirable. One disadvantage of this gel is its possibility of being toxic and non-degradable (Litowczenko et al., 2021).

b) The second category of materials, contrary to the previous case, turns into a gel with a decrease in temperature (Figure 2B). The use of these materials is limited because the solution must have a high temperature before injection, in which biomolecules and sensitive drugs may be damaged (Ward and Georgiou, 2011).

c) In the third category, known as thermoreversible gels, both of these behaviors are seen, that is, with the increase in temperature, first a conversion of sol to gel, and then at higher temperatures, a conversion of gel to sol is observed in them (Figure 2C). The mechanism of gelation in some amphiphile members (substances with both hydrophilic and hydrophobic properties) of this class of materials is that when the temperature rises above the LCST of the gel, the monomers self-assemble due to the polymer-polymer interaction and turn into micelles, after their fraction exceeds a certain limit and the micell packing and knots reach such a level that the micelles cannot move, so the system turns into a gel (Xin and Naficy, 2022).

One of the important formulas in this field is triblock copolymers, the most important part of this family is poloxamer or pluronic with the chemical formula PEO-PPO-PEO. Whose central block is hydrophobic, and the side blocks are hydrophilic. Meanwhile, poloxamer407 or pluronicF127 has the least toxicity and the most use. However, due to the weakness of the cross-links between the micelles, the mechanical properties of this material are weak, and in addition, in a short time, the structure is separated, and release from them happens. In addition, these materials are not biodegradable, and these items can be improved by modifying the structure (Matanović et al., 2014).

To check gelation from a thermodynamic point of view, the formula ∆G = ∆H-T∆S should be referred to. For polymers with LCST, gelation proceeds mainly due to increased entropy, especially in the water component, which reduces the Gibbs free energy. However, in polymers with UCST, the driving force of gelation is enthalpy type. Despite all the mentioned advantages, this method also has its disadvantages. For example, suppose the speed of gelation is high. In that case, there is a possibility of gelation of the substance inside the syringe needle and blocking it during injection, or with slow gelation, the release of these substances has a burst state for a while. The speed of gelation in tissue engineering applications is effective in cell distribution.

In general, in the case of block copolymers such as poloxamer and polymers containing PANIPAAM, to modify characteristics such as gelation time, temperature/pH of gelation, mechanical strength, biocompatibility, it is possible to work on copolymer composition, hydrophobic-hydrophilic balance, two-component block length, weight molecular, polymer architecture. Chenite and co-workers added polyol phosphate salts to chitosan to add temperature-sensitive gelation properties to a pH-sensitive solution and to prepare a biodegradable and biocompatible gel with a good residence time for the delivery of cells and therapeutic proteins (Chenite et al., 2000).

Cho and colleagues proposed a water-soluble chitosan-g-NIPAAm copolymer to treat bladder or gastric reflux during a single-injection endoscopic procedure (without a double-injection system). Human mesenchymal stem cells were placed and differentiated into chondrocytes inside this gel. Lowering the polymer’s temperature from 32°C or LCST allowed the cells to be revived. With the use of this gel, it was possible to study the development of cartilage in the submucosa layer of rabbits’ bladders (Cho et al., 2004).

**FIGURE 3:**
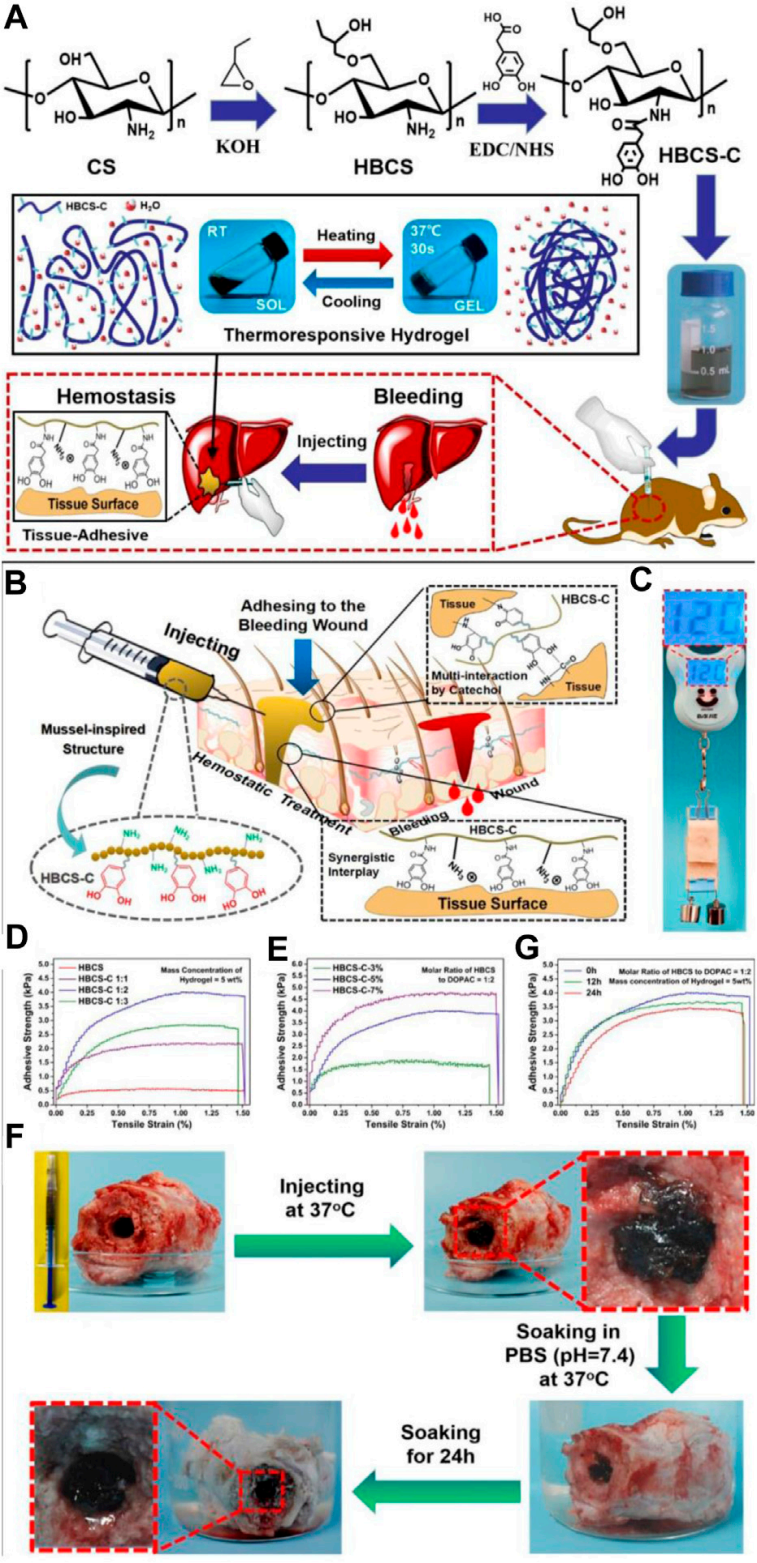
**(A)** HBCS-C thermoresponsive sticky hydrogels shown schematically. **(B–G)** Tissue adhesive behaviors of HBCS-C hydrogels. **(B)** Mussel-inspired HBCS-C hydrogels’ possible tissue-adhesive mechanism is depicted schematically; **(C)** The image demonstrated the HBCS-C hydrogel’s strong adhesive ability on pig skin; lap shear tests of the HBCS-C hydrogels **(D)** at different molar feed ratios of HBCS to DOPAC and **(E)** at different mass concentrations; **(F)** Photos captured the HBCS-C hydrogel’s wet bioadhesive activity and stability in an aqueous environment at 37°C. The HBCS-C hydrogel was still firmly adhered in the hole after 24 h of immersion in PBS, demonstrating its excellent tissue-adhesion and stability; **(G)** Lap shear tests at a different immersing time in PBS. Reprinted with permission from (Shou et al., 2020). Copyright 2020 American Chemical Society.

Shou created the thermosensitive, injectable, tissue-adhesive, biodegradable, biocompatible, and wound hemostasis multifunctional HBCS-C hydrogels (Figure 3). They exhibit an excellent liquid-gel transition at different temperatures because of differences in the hydrophilic-hydrophobic interaction and hydrogen bonds created by the hydroxybutyl groups. The interactions between tissues and catechol/amino groups, which occur often, allow biocompatible hydrogels to cling firmly to tissue surfaces (Shou et al., 2020).

Chung and colleagues prepared Pluronic stereo complex hydrogels linked by D-lactide and L-lactide oligomers for sustained and zero-order release of human growth factor with two mechanisms of diffusion and erosion, which had a linear mass erosion profile. The concentration and critical gelation temperatures were much lower than uncomplexed formulas or pluronic homopolymers. This degradable gel had higher strength than the previous samples, probably due to the spontaneous formation of stereo-complex crystal regions and more cross-links. It was also more resistant to being dissolved in water environments. This gel can be used for gene or cell delivery (Chung et al., 2008).

Yu and colleagues investigated the ability of thermoreversible PLGA–PEG–PLGA for sustained release of PEGylated drugs and used camptothecin as a hydrophobic antitumor model drug (Yu et al., 2008).

Lee and colleagues used a methylcellulose hydrogel to encapsulate spherical aggregates of cells instead of injecting single cells into muscle tissues, which would remain in that area due to their larger size. By using this technique, ECM and intercellular connections were preserved due to not using any proteolytic enzymes during their implantation. In addition, most of the cells were viable; they maintained their aggregate state after injection and maintained their activity by moving to another growth surface. The gel was injected into rabbit skeletal muscle and showed that the aggregates were large enough to stay in the muscle spaces and form a good ECM (Lee et al., 2009).

Choi and co-workers synthesized a polyalanine-poloxamer-polyalanine hydrogel in which the polypeptide end blocks were made using L-polyalanine and D-polyalanine and a mixture of the two and controlled their secondary structure in terms of the β-sheet fraction, which It was effective on the population and nanostructure of the fibrous hydrogel, and as a result, the proliferation and expression of the chondrocyte protein that was encapsulated in it, and it also performed well in their differentiation (Choi et al., 2010).

Hsiao and his colleagues partially substituted chitosan with the hydrophilic carboxymethyl group to increase chitosan water solubility and with the hydrophobic hexanoyl group to increase amphiphilicity. This material was self-assembled and created nanocapsules, which created a gel by combining it with a polyol salt. This degradable substance is used to release therapeutic agents. These substances showed little host inflammation and toxicity on human retinal epithelial cells and had good drug loading (Hsiao et al., 2012).

Chen and his colleagues used hexamethylene diisocyanate to create cross-links in pluronic F127, which increased its mechanical stability and biocompatibility. These nanostructures were used to prepare a composite with a hyaluronic acid matrix. This substance was used to bring the anticancer drug DOX to the zero-order state, which had a negative effect on cancer cell viability and tumor size. In addition, it was shown that the regulation of degradation time could be achieved with the concentration parameter of nanostructures (Chen et al., 2013).

Gelation of temperature-sensitive polymers with increasing temperature can occur due to the rotation of. superparamagnetic iron oxide nanoparticles (SPIONs) in an alternating magnetic field. Zhang and his colleagues proposed using SPIONs in chitosan and β-glycerophosphate hydrogels to deliver *Bacillus* Calmettee Guérin, an immunotherapy drug for bladder cancer (Zhang et al., 2013).

Boustta and his colleagues synthesized Poly (N-acryloyl glycinamide) UCST gel in which the burst release was minimized and could deliver neutral and ionic drugs, especially soluble in an aqueous medium, salts, charged macromolecules, and proteins (Boustta et al., 2014).

### Redox reactions

Utilizing a water-soluble initiator for redox-polymerization. For redox/photopolymerization, synthetic hydrophilic macromers containing acrylate/methacrylate groups were developed. Using similar methods, semi-synthetic macromers derived from natural polymers were studied.

In order to create a hydrogel, functionalized macromers with acrylate/methacrylate groups can be polymerized using free radicals created in water by redox processes. As a redox-initiating system, ammonium persulfate/N, N, N-tetramethyl ethylene diamine (APS/TEMED) or APS/ascorbic acid have typically been employed. The biocompatibility of oxidizing or reducing substances can be a problem in the *in situ* gelling system based on redox reactions. When catechol-containing polymers are oxidized, quinine, an oxidized catechol, may react with the amino groups of the medication absorbed during the gel-forming process. The cysteine groups of the protein medicines can obstruct the thiol-disulfide process. The potential inclusion complex between cyclodextrin and the medication or a portion of the drug should be carefully followed in the event that an inclusion complex forms to create a gel.

Oxidative stress, which is caused by reactive oxygen species (ROS) such superoxide and hydroxyl radicals, has a significant impact on aging and a number of diseases. We created redox polymers with covalent bonds to antioxidants. These polymers, which have the ability to self-assemble, create redox nanoparticles (RNPs) in aqueous media, which limit absorption into healthy cells, build up at regions of inflammation, and successfully fend off diseases caused by ROS. As a result, RNPs are successful in stopping disorders that involve ROS, including cancer, myocardial and brain ischemia-reperfusion injuries, ulcerative colitis, and ulcerative colitis. We created injectable redox gels (RIGs), which, when exposed to physiological circumstances, change from a flowable solution at room temperature to a gel at body temperature. RIGs can be used to reduce localized inflammation, such as those caused by periodontitis. Anti-tissue adhesion sprays used following physical surgery can also contain RIGs.

The electron transport pathway in the mitochondria of healthy cells generates a lot of ROS. Small molecular antioxidants reach healthy cells and their mitochondria, where they have an unfavorable influence on the critical redox processes involved in electron transfer chains. It is impossible to provide enough LMW antioxidants because ROS are “double-edged swords” in this way; these antioxidants have a small or non-existent therapeutic window. We created a different type of antioxidant nanomedicine. This is a covalent compound of a typical antioxidant and a metabolizable, moderately-molecular-weight polymer. An amphiphilic block copolymer with a self-assembling property was covalently conjugated with a nitroxide radical (TEMPO) with catalytic ROS scavenging capabilities [8]. In an aqueous solution, the amphiphilic redox polymer self-assembles to produce nanoparticles (Ko et al., 2013; Nagasaki, 2018).

Numerous cardiovascular illnesses are impacted by nitric oxide (NO), but its therapeutic use is constrained by its short half-life and low absorption. Overproduced reactive oxygen species (ROS) quickly react with endogenous NO in inflamed tissues, lowering its bioavailability. When there has been a myocardial infarction, the infiltration of inflammatory cells like macrophages and the ensuing inflammatory response are crucial for tissue repair. However, if the inflammatory response becomes excessive, it will slow wound healing and lead to persistent heart failure.

The inducible NO synthase (iNOS) enzymatic process can produce NO by using L-Arg as a substrate since these activated macrophages lead to a considerable rise in iNOS expression and activity.

Vong et al. (2018) designed a formulation based on PArg-PEG-PArg and PMNT-PEG-PMNT coupled with PAAcs for preventing and treating cardiovascular diseases, in which the polyion complex (PIC) micelle was formed in the flower-type and turned into a gel by changing the temperature during the injection. This substance spread homogeneously and remained in the myocardial tissue, releasing NO and scavenging overproduced ROS. This redox-responsive gel caused angiogenesis, reduced infarction size, and good heart function after infarction.

Cao and his colleagues produced a thermally reversible gel that contained self-assembled peptide nanofibrils and PNIPAM, and when the temperature passed from the LCST of PNIPAM, the globules formed from it acted as a cross-linker of the fibrils and a gel was created. The release rate can be adjusted by the concentration of the peptide due to the affinity between them. To remove this material, the temperature can be reduced below the LCST and removed from the site with slight mechanical pressure. This gel can be used in drug delivery, and due to its fibrillar structure similar to ECM, it can potentially be used for tissue engineering (Cao M. et al., 2019).

**FIGURE 4:**
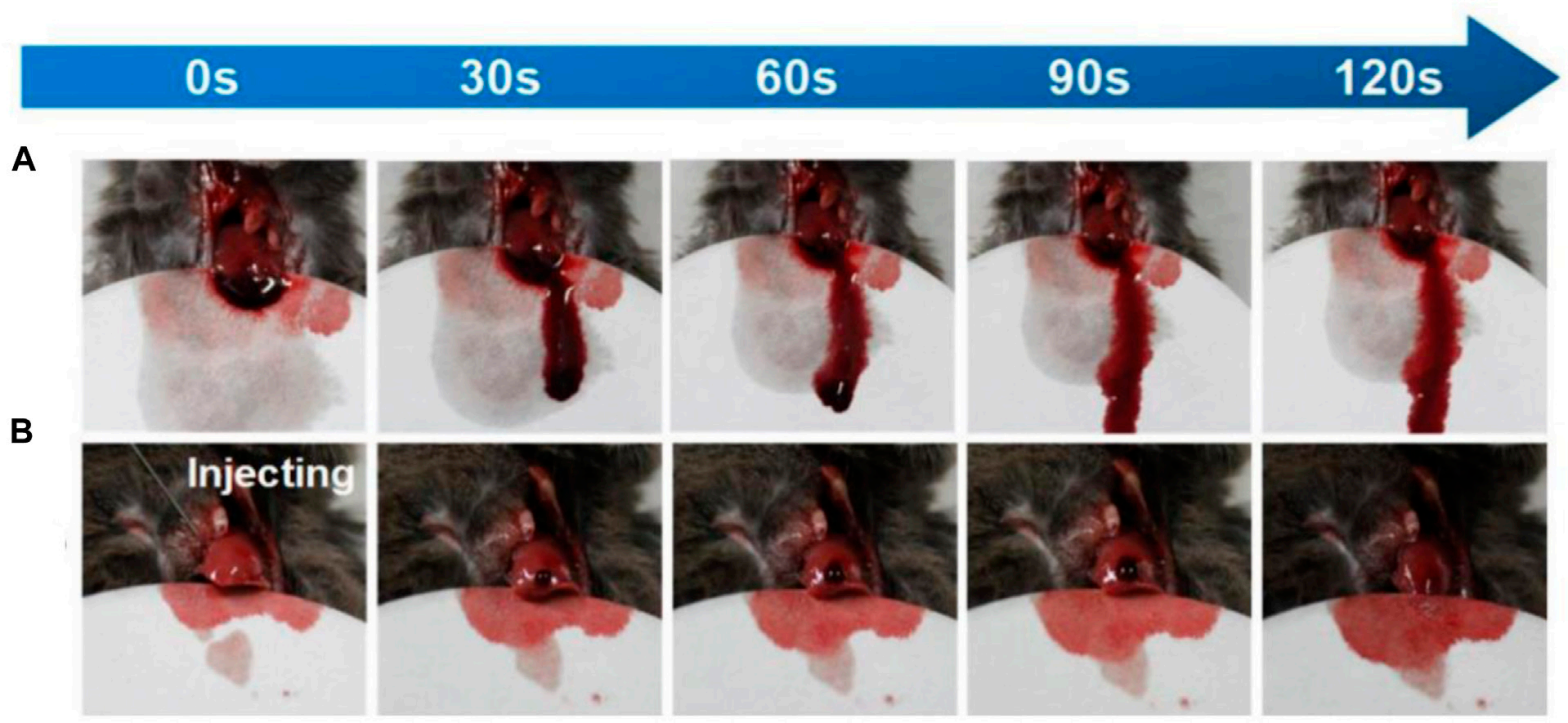
The amount of bleeding in the mouse liver over time **(A)** without and **(B)** with gel. Reprinted with permission from (Shou et al., 2020). Copyright 2020 American Chemical Society.

Shou and colleagues used a chitosan-based gel for the hemostasis of bleeding wounds. Unlike the previous samples, this gel had very good tissue adhesion, which resulted in excellent homeostatic properties, and was biocompatible and biodegradable (Figure 4) (Shou et al., 2020).

Tan and colleagues developed a PLGA-PEG-PLGA-based nanocomposite gel containing metal-organic frameworks, DOX, and the anti-angiogenic agent celecoxib for treating oral cancer, which has high drug loading capacity, sustained release, and pH-responsiveness of both types of drugs was one of its benefits (Tan et al., 2020).

### pH

**FIGURE 5:**
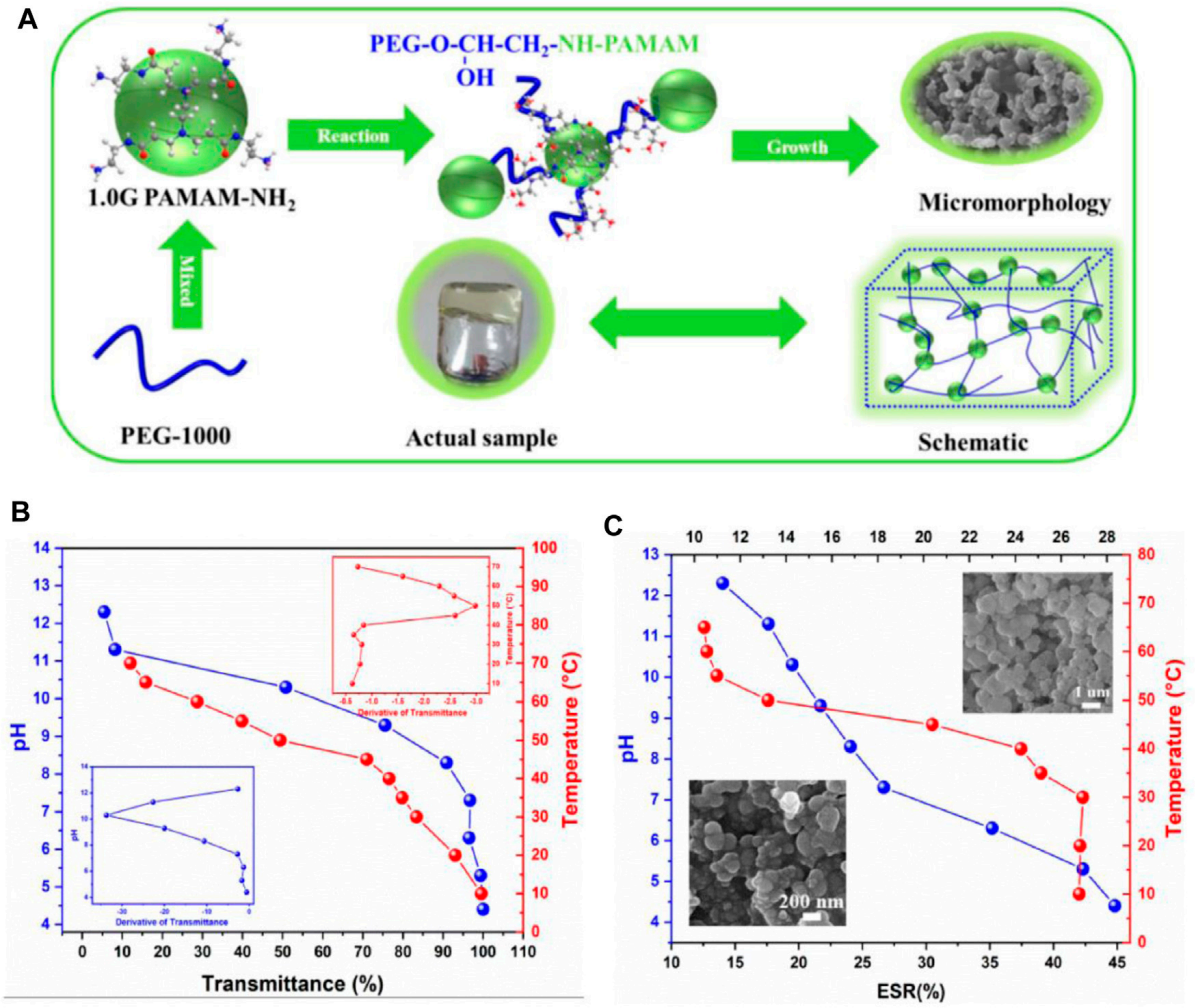
**(A)** Diagram of PEG/PAMAM hydrogel gelation. **(B)** Transmittance and phase transition temperature and pH curves of PEG/PAMAM hydrogels. **(C)** Equilibrium swelling ratio curves of PEG/PAMAM hydrogels under different temperatures and pHs (Liu X. et al., 2022).

pH stimulus is mostly used to induce volume change and drug release in gels, but cases of gelation induction have also been reported by applying this stimulus (Figure 5). Chitosan, the N-deacetylated derivative of chitin and the only natural cationic polysaccharide, is known to gelate due to pH change. This substance is a polybase that is protonated in a mild acid, and the dangling primary amine groups receive a positive charge; that is, they are converted from the formula -NH_2_ to -NH_3_^+^, which creates a repulsive force in it and causes its dissolution and transformation to sol. Nevertheless, it turns into a gel again after crossing the Pka point, removing the positive charges and creating a hydrogen bond between the amine groups (Yuan et al., 2022). This substance has an acidic pH in the sol state that causes inflammation in the host tissue, which subsides with the degradation of the gel. To increase the strength of polymers in general, it is possible to use hydrophobic modification and polymer blends. The gel’s strength depends on the polymer’s crystallinity and, as a result, the porosity of the gel (Podgórski et al., 2020). Due to the acidic pH of inflamed areas or tumors, these gels can be used in cancer treatment with chemotherapy, gene therapy, or immunotherapy. For example, nanogels containing anticancer drugs can be injected intravenously so that they accumulate in the tumor area and become a bulk gel, which can be designed with target ligands for this purpose. Formation of gel and then releasing the drug in the target area helps to prevent systemic side effects. Gels of other categories, for example, thermo-responsive, can be modified with ph-responsive groups and create a dual-responsive gel (Huang et al., 2021). To create more intermolecular forces and enhance gelation in chitosan, Chiu and colleagues modified it with a hydrophobic palmitoyl group and obtained N-palmitoyl chitosan (NPCS). In which gelation occurs in a narrow pH range of 6.5–7. This non-toxic and degradable gel can be used for drug and cell delivery (Chiu et al., 2009).

Li and colleagues reacted to PEG derivatives and α, β-polyaspartylhydrazide during the injection. The resulting gel was able to transform into a sol in the tumor tissue with an acidic pH, release the anticancer drug DOX, and return to the gel state with an increase in pH. The resulting material was biocompatible and biodegradable and was tested on mice with human fibrosarcoma (Li et al., 2015).

**FIGURE 6:**
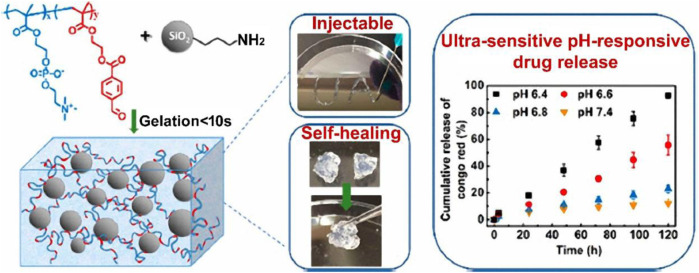
Illustration of injectable and self-healing nanocomposite hydrogels with ultrasensitive pH-responsiveness and tunable mechanical properties. Reprinted with permission from (Wu et al., 2020). Copyright 2020 American Chemical Society.

The work of Wu et al. (2020) Aldehyde-functionalized polymers and amine-modified silica nanoparticles interact through Schiff base interactions to create injectable, self-healing, and pH-responsive nanocomposite hydrogels (Figure 6). The hydrogel gelates in 10 s, is injectable and has quick self-healing capabilities. Additionally, the hydrogel demonstrated amazing stability in physiologically neutral conditions, but a mildly acidic environment caused a quick gel-sol transition, which was suitable for regulated drug delivery. The hydrogel’s pH responsiveness was extremely sensitive; when subjected to a 0.2 pH shift, mechanical properties, hydrolytic breakdown, and drug release behaviors all changed significantly.

Moreover, the composition of the hydrogel might easily alter its mechanical and pH-responsive capabilities. Cytotoxicity testing on human dermal fibroblast cells (HDFa) demonstrated good biocompatibility.

**FIGURE 7:**
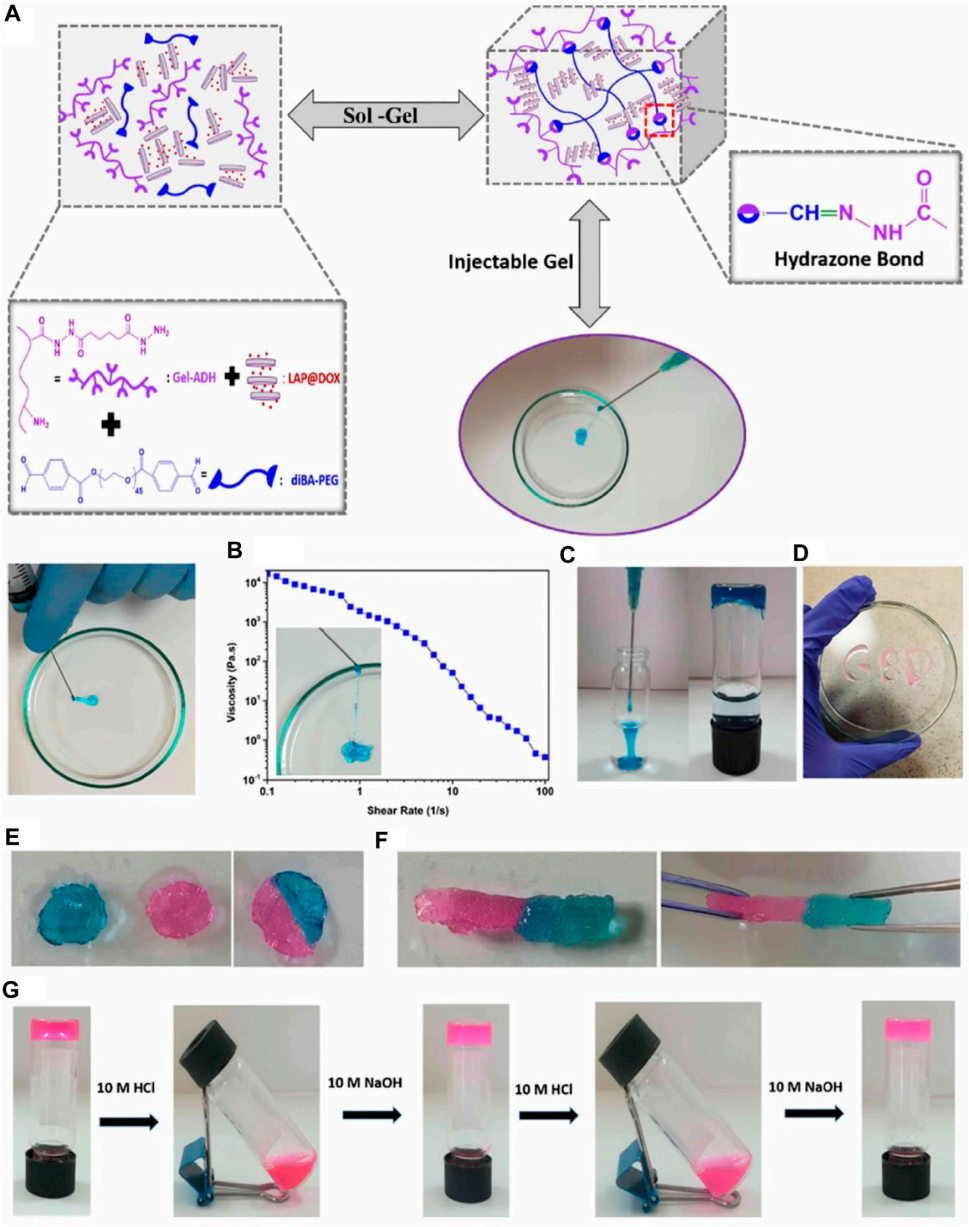
Injectability of the hybrid hydrogel: **(A)** hybrid hydrogel was injected through a 21-gauge needle, **(B)** diagram of the apparent viscosity rheology as a function of the shear rate of the hybrid hydrogels. **(C)** Injection of the Gel-ADH/diBA-PEG/LAP hybrid hydrogels into the PBS buffer and the photograph of the formed hydrogel into the PBS solution **(D)** Gel-ADH/diBA-PEG/LAP hybrid hydrogels injected from a 21-gauge needle onto a Petri dish. Self-healing behavior of the Gel-ADH/diBA-PEG/LAP hybrid hydrogels: the hybrid hydrogel was prepared in two colors, that is, pink and blue, separately, and these two-hybrid hydrogels were allowed to make contact with each other **(E)** photograph of the self-healed hydrogel after 1 h, and **(F)** stretching behavior of the self-healing hydrogel after 1 h **(G)** pH-dependent decomposition−composition behavior of the hybrid hydrogels. Reprinted with permission from (Cimen et al., 2021). Copyright 2021 American Chemical Society.

Hybrid hydrogels that are injectable, self-healing, and pH-responsive are prospective biomaterials for the gradual release of therapeutic chemicals during the course of cancer treatment. Cimen et al. (2021) Hydrazone linkages were produced using aldehyde- and hydrazide-functionalized gelatin (Gel-ADH) and PEG (diBA-PEG) polymers. They have developed an injectable hybrid hydrogel that responds to pH (Figure 7). By assimilating the resulting Pregele with Laponite (LAP) nanodiscs loaded with the anticancer medication doxorubicin (DOX) during the gelation process, an ADH/diBA-PEG/LAP@DOX hybrid hydrogel was created. The hybrid hydrogel’s observed gelation time was 80 s, and the finished product had outstanding injectability and quick self-healing. The injectable hybrid hydrogels had a very efficient and pH-dependent long-term drug release profile. Human breast cell line (SVCT) and endothelial cell line (HUVEC) were used to assess the hydrogel’s components for biocompatibility. The hybrid hydrogel’s components are entirely biocompatible and even encourage cell growth. In addition, human breast cancer cell lines and triple-negative breast cancer cell lines were used to test the hydrogels’ cytotoxicity.

### Ion

Certain polyelectrolytes, such as alginate and pectin, which become anions by dissolving in water and, for example, loses H of its carboxyl group, are connected by 2- or 3-valent counterions such as Ca_2_^+^ and become a gel. In this way, CaCl_2_ solution or an aqueous slurry of Ca salts is injected into the desired area with a polymer solution using a two-syringe applicator. Adjusting the cross-link density and, consequently, the gel’s mechanical properties can be achieved by adjusting the number of counter ions. However, the excessive concentration of opposite ion causes toxicity and damage cell life and therapeutic effect. Therefore, this parameter has an optimal value. To avoid increasing the Ca^2+^ concentration too much, salts of this ion with lower solubility can be used. For example, α-TCP releases Ca ions in water. Another solution is to use calcium-free gels.

Alginate and pectin do not have good cell adhesion, but this issue can be solved with some measures. In addition, the degradation of alginate gels occurs slowly because hydrolysis or enzymatic degradation is not performed on these materials. However, hydrolysis becomes possible with partial oxidation, and the degradation rate increases (Van Tomme et al., 2005). Except for ionic cross-linkers, charged small molecules have also been used for this type of gelation. These agents create similar bonds, but there are differences in penetration speed, etc. Of course, there are other types of ionic gelation, for example, the combination of two polyelectrolytes with opposite charges, which creates a polyelectrolyte complex. Gelation by ion and pH stimuli is performed with a mass transfer mechanism, which is slower compared to temperature gelation through a heat transfer mechanism, and as a result, the problem of rapid gelation and syringe blockage does not occur in these methods.

Tomme and colleagues mixed dispersions of oppositely charged dextran gel microspheres. The gel network was degraded under low pH and high ionic strength. This gel was used in drug delivery and tissue engineering (Van Tomme et al., 2005).

Douglas and colleagues prepared a composite gel containing pectin derived from citrus peel and apple pomace and calcium-rich bioactive glass particles derived from the gel. These glass particles, releasing calcium ions for pectin gelation, caused an antibacterial property as well as mineralization of the gel in the simulated biological environment, which the last item was related to the particle size. Glass with smaller particle size caused a higher ion release rate and thus higher gelation speed. The distribution of glass particles in the gel was not uniform. This gel could be used in bone tissue engineering and showed good cell compatibility with MC3T3-E1 osteoblast cells (Douglas et al., 2018).

Wang J. H. et al. (2021) prepared a hybrid gel with interpenetrate network composed of pectin methacrylate and methacryloyl gelatin for skin hemostasis. In this gel, cross-links were created by light and ions. The high porosity of the gel network caused blood absorption and coagulation at high speed. The characteristics of the gel were easily adjustable and easily removed from the site.

## Biological stimuli

### Enzyme

Enzymes are the only important biological and internal factors that can cause gelation. Some protein hydrogels, such as fibrin, collagen, and gelatin hydrogels, can be formed with transglutaminase enzymes, known as bioadhesives; This enzyme creates an isopeptide chemical bond, which is an amide bond between two protein molecules. This bond is highly resistant to proteolysis and has good stability in the body. FactorXIIIa enzyme is one such type of enzyme that catalyzes the gelation of fibrin hydrogels. Another enzyme widely used in the gelation of polysaccharide-based polymers is the horseradish peroxide (HRP) enzyme, which works in the presence of an oxidant such as H_2_O_2_ as a substrate. This enzyme is stable, has an easy purification process, and creates strong gels. This type of gelation is widely used in tissue engineering applications and interaction with cells due to the biocompatibility of the reaction. In addition, the reaction is carried out at neutral pH and mild temperature, and due to the specificity of the function between the enzyme and the substrate and the absence of side reactions, no toxicity is caused.

Sanborn and his colleagues developed a system consisting of phospholipid vesicles containing calcium, thrombin, and factorXIII along with a four-armed PEG with a 20-mer peptide derived from fibrin γ chain attached to an N-terminal cysteine. By changing the temperature at 37°C, the liposomes changed phase, and calcium was released from them, which, along with thrombin, activated factorXIII, which catalyzed the gelation and created a strong and elastic gel that It could not be cut under oscillating cutting and of course it was degradable. This type of gelation was inspired by the cross-link that is done in blood coagulation. This gel is used in drug and gene delivery and tissue adhesion (Sanborn et al., 2002).

Mchale and his colleagues performed genetic engineering on an elastin-like polypeptide, which turns into a gel in the presence of tissue transglutaminase (tTG). This gel can differentiate cells and promote chondrogenesis and control mechanical properties. This material can be used both with and without cells. If used with cells and encapsulation of chondrocyte cells and bioactive factors, the cells had good viability, the phenotype of the cells was maintained, and the increase in the mechanical integrity of the gel indicated ECM deposition. The gel showed good cell penetration if used without cells (McHale et al., 2005).

Jin and co-workers found the formation of dextran-tyramine conjugated gels by HRP enzymes in the presence of H_2_O_2_, which was used for cartilage tissue engineering. Dextran molecular weight and tyramine substitution degree were effective on gelation time and storage modulus. Chondrocyte cells had good survival and proliferation in this gel, maintained their spherical shape, and produced a cartilage-specific matrix, namely GAG and type II collagen. GAG accumulation gradually increased inside the gel (Jin et al., 2010b).

**FIGURE 8:**
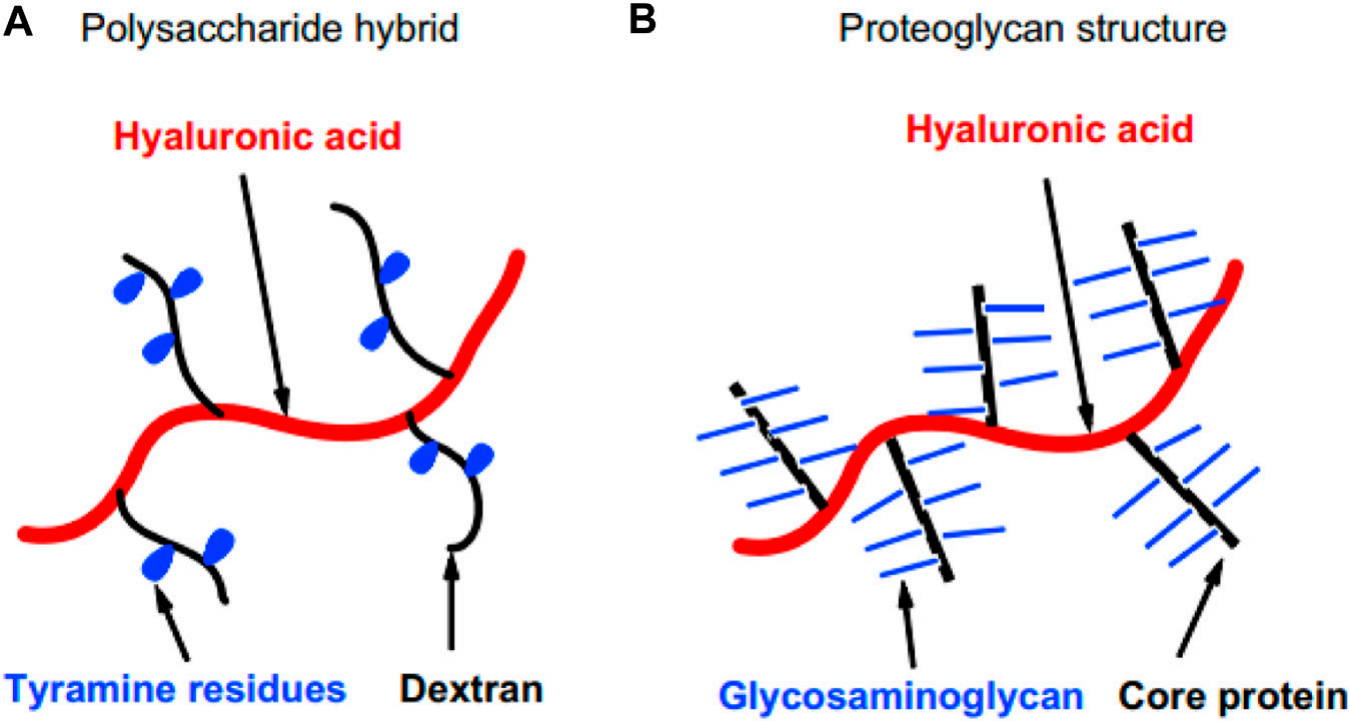
Schematic illustration of **(A)** the molecular structure of the material and its similarity to **(B)** proteoglycan of cartilage ECM.

The same group developed a hyaluronic acid gel grafted with a dextran-tyramine conjugate (Figure 8A) for bone tissue engineering application. That the HRP enzyme, in the presence of H_2_O_2,_ accelerated the establishment of the cross-link of tyramine. The use of this gel led to good cell survival, proliferation, matrix production (collagen and GAG), and differentiation. The proteoglycan of the natural cartilage ECM inspired the molecular structure of this gel (Figure 8B). The gel was degraded in the presence of hyaluronidase enzyme. The degree of tyramine substitution and polymer concentration was effective on gelation time, equilibrium swelling, and storage modulus (Jin et al., 2010a).

Ren and co-workers constructed a glycopolypeptide by conjugating poly (g-propargyl-Lglutamate) with azide-modified mannose and 3-(4-hydroxyphenyl) propanamide, which HRP and H_2_O_2_ gelled. The concentration of these two was effective on the gelation time, storage modulus, and degradation and swelling time. This gel had useful features such as fast gelation and degradability, and when used with cells, it caused cell survival, cell compatibility, phenotype preservation, cell proliferation, and type II collagen production. In the construction of this gel, natural glycopolypeptides and glycoproteins were mimicked, and their function was similar to the proteoglycans of natural cartilage ECM (Ren et al., 2015).

Inspired by platelets blood coagulation and using serotonin, Zhang et al. (2021) prepared a gel for hemostasis and then wound healing. Chondroitin sulfate was also present in that gel, which regulated cell behavior and wound healing. The HRP enzyme did gelation in this material, and it caused biocompatibility, proper cross-linking, and degradability. Table 1 represents examples of gels formed by enzymes in the literature.

**TABLE 1:** Examples of gels formed by enzymes in the literature.

Enzyme	Gel	Example	Ref
transglutaminase	Protein hydrogels	a peptide derived from fibrin and factorXIII	Sanborn et al. (2002)
		elastin-like polypeptide	McHale et al. (2005)
HRP	Polysacharide hydrogels	dextran-tyramine	Jin et al. (2010a)
		hyaluronic acid-g-dextran-tyramine	Jin et al. (2010a)
		poly (g-propargyl-Lglutamate)-azide-modified mannose-3-(4-hydroxyphenyl) propanamide	Ren et al. (2015)
		Serotonin-Chondroitin sulfate	Zhang et al. (2021)

## Exogenous stimuli

External stimuli are applied to induce gelation from outside the body.

### Photo

This stimulus is used to form and destroy the gel network. The formation of the network by this method under UV or visible light includes three stages initiation, propagation, and termination. At first, there are monomers or oligomers of the substance and a substance called photoinitiator, which uses free radical type photoinitiators with a controlled concentration that does not cause significant toxicity. Usually, UV light irradiation causes the excitation and breaking of a specific bond in the photoinitiator molecule and the formation of a free radical (a particle that has at least one unpaired valence electron), which this free radical separates the double bonds of the main chain of monomers, and create new free radicals. In the propagation stage, which occurs rapidly, these continue the same process on the monomers around them, and the interaction between the two radicals creates a long polymer chain with chemical bonds. This type of polymerization is widely used in tissue engineering (Jiang et al., 2022). Having control over the time and place of radiation is one of the outstanding advantages of this method, and for this reason, it is possible to create micropatterns on the surface and adjust cell behavior, including adhesion, migration, and differentiation in the field of tissue engineering or setting the release rate in different places in the field of therapeutic agent delivery. Of course, these techniques are mostly used for pre-formed gels and not for injectable gels.

**FIGURE 9:**
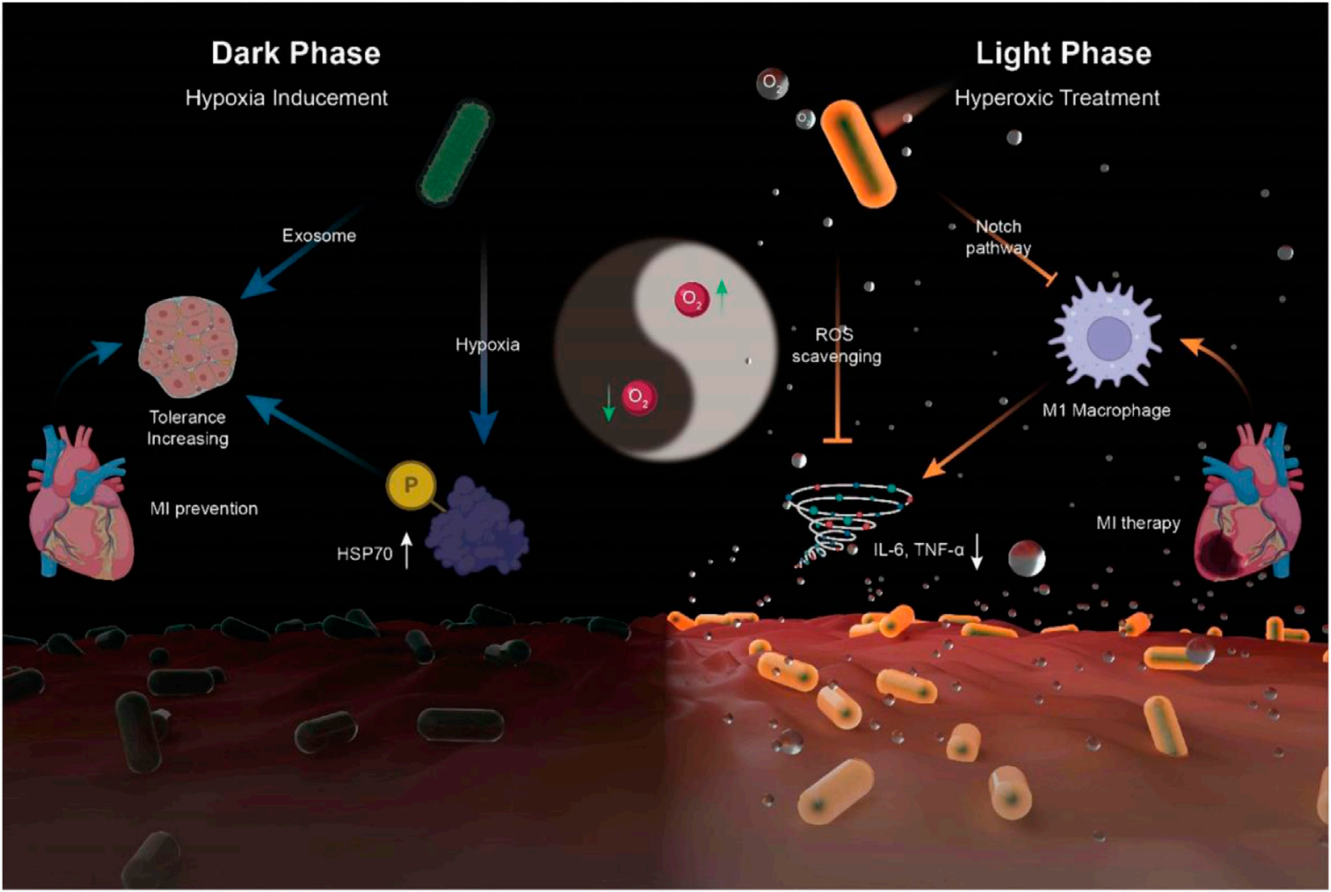
Synthesis of UCCy@Gel for acute MI prevention (dark phase) and therapy (light phase): the UCCy@Gel consumes oxygen *via* respiration to generate a hypoxic microenvironment, resulting in the upregulation of HSP70, which enhanced the tolerance of CMs for MI (left); Under 980 nm NIR irradiation, the UCCy@Gel releases photosynthetic oxygen through UCL to inhibit macrophage M1 polarization and downregulated proinflammatory cytokines IL-6 and TNF-α to repair myocardial injury (right) (Liu Y. et al., 2022).

In addition, in this method, the gelation speed is higher than other methods, such as gelation induced by temperature, due to the high energy of UV light and the light speed compared to heat transfer in bulk. The high rate of polymerization can cause a great increase in temperature and damage to cells and biomolecules; In this regard, to control the speed of gelation, it is possible to change the intensity of light or the type and concentration of photoinitiator or to irradiate light at intervals during gelation, and also to adjust the amount of reactive double bonds. A long irradiation time leads to an increase in the cross-link density in the gel. It is effective on the gel’s mechanical properties, swelling or degradation, and cargo release. It limits the diffusion of nutrients and O_2_ and the migration of encapsulated and host cells. This problem can also be solved by surface polymerization, not and not by bulk (Liu Y. et al., 2022). Liu et al. reported a hydrogel-coated upconversion cyanobacterium nanocapsule (UCCy@Gel) for MI prevention and therapy (Figure 9): upconversion nanoparticles (𝛽- NaErF 4@NaLuF4 nanocrystals, UCNPs), which could absorb deeper tissue penetrable near-infrared (NIR) photons and emit shorter wavelength photons (upconversion luminescence, UCL), was conjugated to the surface of cyanobacteria. Besides, a methacrylate outer hydrogel layer was formed to enhance stability and provide stable tissue adhesion. When necessary, through the excitation of 980 nm NIR light, a blend of visible light emitted by UCNPs were used for photosynthesis in cyanobacteria to achieve appropriate oxygen liberation. By this means, a flexible NIR-controllable oxygen-modulating cyanobacterium nanocapsule could be realized for both MI hypoxic prevention and local oxygen therapy (Liu Y. et al., 2022).

In this method, there is a possibility of damaging DNA and cell function and changing gene expression due to the presence of UV light and free radicals. On the other hand, the penetration depth of UV light in the body tissue is low, and therefore this method is mostly used for subcutaneous applications and at shallow depths of the body; Of course, the use of laparoscopic radiation tools is a possible way to use this method for areas that are far away from the surface of the skin. The limited penetration of UV also causes a rigid gel not usually obtained, and to solve these problems, high-intensity visible light can be used, which has deeper penetration and causes less damage to the tissues. Elisseeff and his colleagues realized the possibility of penetration of UV and visible light in living tissues such as skin and polymerization under indirect light irradiation and investigated the potential of this material for the application of cartilage tissue engineering and the release of a protein drug, which led to the creation of collagen and proteoglycan in the first application (Elisseeff et al., 1999).

Such gels can be obtained by modifying materials with functional groups capable of cross-linking by light, such as acrylate groups and their derivatives. For example, Nettles and his colleagues synthesized a methacrylate hyaluronan gel based on natural hyaluronan and thus plays an important role in the development and function of cartilage and has good mechanical properties. Chondrocyte cells were encapsulated in this gel and kept their spherical shape. This resulted in the accumulation of a large amount of matrix and infiltration of cells from the gel into the defect, which resulted in a good integration (Nettles et al., 2004). Light-induced gelation can occur in temperature-sensitive solutions in which photothermal agents are present. In this way, photothermal agents produce heat with light radiation, and as a result, the gel is formed. In this context, Kawano and his colleagues designed a system consisting of several PNIPAM nanogels, each of which contained a gold nanorod, which was systemically injected and circulated, and then by absorbing NIR light at the target site, the hydrophobic surface of the nanorod changed, formed aggregates and accumulated in that place, and then produced heat and caused the reversible phase transformation of PNIPAM (Kawano et al., 2009).

Wu and his colleagues designed a system containing graphene oxide nanosheets and a peptide consisting of glycine-alanine (GA) repeats flanked by a motif for binding to the former and a motif for cross-linking by light for drug delivery (Wu et al., 2014). This gel was self-assembled by creating a β-sheet from the juxtaposition of GA components. Then, a cross-link was formed by light irradiation and with the help of a ruthenium catalyst. The resulting gel had high mechanical stability and a low erosion rate. The radiation of NIR light and the generation of heat by the graphene oxide sheets caused the β-sheets of the structure to unfold partially, the interaction between the two components of the structure was weakened, and the drug contained in it was released (Wu et al., 2015).

Qi and his colleagues extracted sericin from silk industry waste and meta-acrylate it, which turned into a gel in the presence of UV. By changing the degree of network modification, the mechanical properties and degradation speed were adjusted, which is necessary for different needs. This biocompatible gel was used for cartilage tissue engineering, and the chondrocyte had good adhesion and growth. It also had a photoluminescent property that made its location traceable at any moment. This gel was biomimetic and, for example, resulted in the accumulation of cartilage-specific ECM compounds or the expression of more of its genes (Qi et al., 2018).

Lim and co-workers used visible light in the 400–450 nm range for the gelation of Gel-MA by ruthenium and sodium persulfate photoinitiators. Despite having comparable properties to gels created by conventional primers, this gel has fewer side effects on the survival and metabolic activity of articular chondrocyte cells, causes more glycosaminoglycan formation, cells show a higher re-differentiation ability in it. Moreover, the light has a higher penetration depth, which makes possible the formation of gels with a higher thickness (Lim et al., 2019).

Chang et al. (2021) used the combination of methacryloyl gelatin and phenyl isothiocyanate-modified gelatin and developed a gel for rapid hemostasis of oral and dental wounds. The resulting gel has biological activity properties due to the presence of GelMA and good toughness due to the presence of Gel-Phe.

**FIGURE 10:**
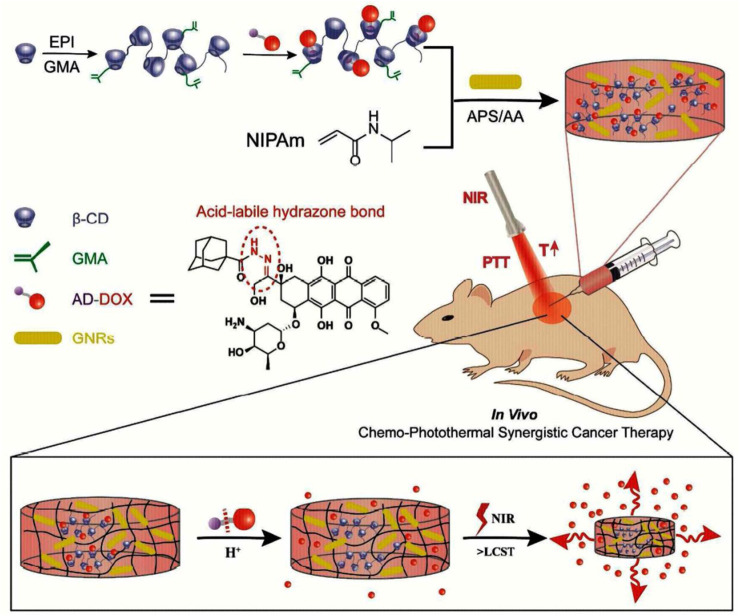
Formation of injectable, NIR/pH-responsive nanocomposite hydrogel and chemo-photothermal synergistic cancer therapy. Reprinted with permission from (Xu et al., 2017). Copyright 2017 American Chemical Society.

An injectable, near-infrared (NIR)/pH-responsive poly (NIPAm-co-MPCD)/GNRs nanocomposite hydrogel was created using the copolymerization of N-isopropyl acrylamide (NIPAm) and methacrylate poly-cyclodextrin (MPCD)-based macromer. This hydrogel could be used as a long-acting implant for chemophotothermal synergistic cancer (Figure 10). The hydrogel had excellent gelation characteristics, NIR responsiveness, and acceptable mechanical and swelling properties. A doxorubicin prodrug modified with adamantane and hydrophobic acid-labile was successfully loaded into the hydrogel by the host-guest interaction. The hydrogel showed prolonged drug release and could maintain a modest, constant flow of DOX for over a month. The acid-labile hydrazone link between DOX and adamantyl groups was seen to break in an acidic environment, revealing the pH-dependent release of DOX from nanocomposite hydrogels. The release of DOX from the hydrogel network that was accelerated by NIR irradiation was controlled by the hydrogel network collapse brought on by the photothermal action of GNR. *In vitro* cytotoxicity experiments on nanocomposite hydrogels showed their good biocompatibility and photothermal effect.

Additionally, the mouse model study showed good tissue biocompatibility. The nanocomposite hydrogels’ capacity to deliver chemo photothermal synergistic therapy with fewer adverse effects was proven by the *in vivo* anticancer test. So, as a long-term drug delivery system, this injectable, NIR/pH responsive nanocomposite hydrogel showed great promise (Xu et al., 2017).

### Shear stress

These materials are known as shear-thin and self-heal materials. Applying shear stress during injection reduces their viscosity; they become an injectable sol, and after the injection is completed and the shear stress is removed, they repair themselves and return to the gel state. These materials are a subset of self-healing materials in which the self-healing process occurs by a stimulus and not by itself. These materials have shape memory characteristics, meaning they leave their original shape in memory and return to their original state after changing the shape. The components of self-healing materials are placed next to each other with reversible physical links, and if a crack is formed in them, it will be repaired by itself, which increases the lifetime of these materials. This method also has a very good gelation speed, so the problems of fast and slow gelation mentioned in the temperature section do not exist for this method. Another advantage of such gels is that they reduce the effect of shear force on cells.

The network structure of the hydrogel in these cases comprises stimuli-responsive moieties that permit prompted and reversible conversion from hydrogel to sol or solid states. For instance, metal ion cross-linked poly (acrylic acid) (pAAc) forms a metal–carboxylate hydrogel that exhibited a light-induced transformation into the sol state. Likewise, a pH-sensitive hydrogel was synthesized using the interaction between the polymer chains functionalized with catechol and boronic acid moieties. Under alkaline conditions, a three-dimensional hydrogelator network was generated by the crosslinking of polymeric chains with boronate–catechol complexes, whereas upon acidification of the hydrogel phase separation took place as a result of the hydrolysis of the boronate ester. Furthermore, the mechanical properties of stimuli-sensitive hydrogels and nanogels can be tuned. The covalent crosslinking of polymeric chains with various bridging molecules or with stimuli sensitive crosslinkers produces stable hydrogels. The dissociation of the crosslinker using various stimuli decreases the stiffness of the hydrogel. Hence by the reversible association and dissociation of the stimuli-sensitive crosslinkers, the hydrogels can be reversibly swapped between upper and lower rigidity (Roy et al., 2022).

Another significant and readily measurable method to represent the self-healing ability is the dynamic rheology test. The amplitude sweep measurement was performed firstly in order to ascertain the linear viscoelastic region of the hydrogel and find out the critical strain value required to break the hydrogel network (Chen et al., 2020). An important factor for injectability from the rheology data is whether the hydrogel is shear-thinning. Shear-thinning hydrogels experience decreases in viscosity upon application of shear, which are enabled by reversible cross-linking mechanisms. Hydrogels with a lower viscosity and lower storage and loss moduli are typically easier to inject than hydrogels of a high viscosity and high storage and loss moduli (Chen et al., 2017).

These materials have many potential applications in the field of tissue engineering. Yan and colleagues studied how shear-thinning and self-healing occur in β-hairpin peptide-based gels. During the injection, the network was divided into gels larger than 200 nm, which penetrated quickly after the shear was completed, and thus the gel was restored. It was also found that the period and speed of shear are effective on the speed of repair and the stiffness of the gel afterward, and in addition, the injection has little effect on cell viability and distribution (Yan et al., 2010).

The repair process in some materials can occur by a mechanism called Dock and Lock. In the complex, the component derived from the R subunit of the protein kinase A family (which can exist in two types, I and II, in PKA isozymes, and each type has two isoforms, *α*, and *β*) dimerizes during the docking phase. And then another component derived from A-kinase anchoring protein that anchors them with high specificity is called the lock stage.

Lu and his colleagues conducted research in this field in which the primary component was attached to both ends of a polypeptide, and the secondary component was attached to the ends of a 4- or 8-arm PEG cross-linker by mixing the two a strong gel formed with physical links between two components. This gel was used to deliver stem cells and large molecules during erosion, and it had a high percentage of survival and compatibility for the cells. The gels’ mechanical properties and erosion speed could be adjusted with the peptide sequence of the second component, the concentration and ratio of each component, and the number of peptides in the cross-linking polymer. Adequate gelation speed, homogeneous encapsulation of the cargo, and repeatability of the reaction were among the advantages of this system (Lu et al., 2012).

Zhang and his colleagues created a stable ferrofluid by dispersing Fe_3_O_4_ nanoparticles in a chitosan solution, which was mixed with an organic solution of PEG to form a gel by creating a Schiff-Base dynamic side bond between the two. The link was broken during the injection and then repaired in this material. Collecting parts for repair could be done by magnetic force. Also, by magnetic conduction, the gel could pass through the channels by changing its shape while maintaining its integrity (Zhang et al., 2012).

Wang and his colleagues prepared a material based on elastin-like protein (ELP) and hyaluronic acid, in which the formation of the secondary network of ELP by changing the temperature during injection reduced the erosion rate and long-term stability. This gel provided good mechanical protection of stem cells during injection, had good cell adhesion, and was degradable (Wang et al., 2017).

Qu and his colleagues developed a shear thin-self-heal hydrogel based on chitosan and PEG for the treatment of liver cell cancer, which was established between the two components of the Schiff-base bond, which was dissociated by changing the pH, the gel was degraded, and as a result, the DOX drug content was released. The drug-free gel was non-toxic to L929 and HepG2 cells. Gelation time, storage modulus, and degradation were adjustable with cross-linker concentration (Qu et al., 2017).

**FIGURE 11:**
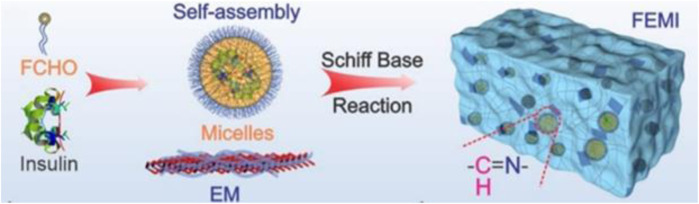
Components of the gel (Wang et al., 2020b). Reprinted with permission from (Wang et al., 2020b). Copyright 2020 American Chemical Society.

Wang and colleagues developed a diabetic wound-healing gel (FEMI) susceptible to multidrug-resistant bacterial infection, hyperglycemia, and an oxidative environment. They used MnO_2_ nanosheets coated with ε-polylysine (EPL) and self-assembled micelles of Pluronic F127 aldehyde containing insulin (FCHO), which formed a Schiff-base bond between them (Figure 11). During reaction $H_{2}O_{2}+MnO_{2}\to H_{2}O+O_{2}+{Mn}^{2+}$ , MnO_2s_ nano-enzymes continuously supplied O_2_ by converting endogenous H_2_O_2_ to O_2_ while reducing the intensity of environmental oxidation. The pH- and redox-responsive gel had long-term insulin release with temporal and spatial control in oxidative and acidic skin conditions. Coated nanoplates in the gel and their interaction with the matrix caused the material’s rapid gelation and stable rheological properties. Fast gelation and tissue adhesion properties caused a good hemostatic application in the gel. Also, the presence of ε-polylysine with a positive charge and nano-enzymes brings antimicrobial properties to the gel. This gel accelerated cell proliferation, facilitated angiogenesis, increased granulation tissue formation and ECM deposition, re-epithelialization, and diabetic wound healing (Wang et al., 2020b).

**FIGURE 12:**
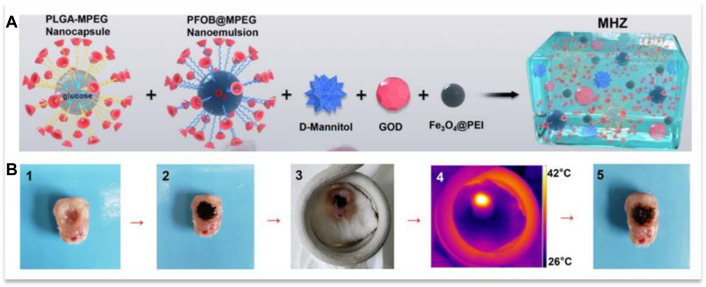
**(A)** the components of the gel and **(B)** its penetration into an isolated tumor during the application of a magnetic field. Reprinted with permission from (Wu et al., 2019). Copyright 2019 American Chemical Society.

Wu and his colleagues developed a gel containing PLGA nanocapsules containing glucose and perfluorooctyl bromide nanoemulsion containing O_2_, which were connected to α-CD through methoxy polyethylene glycol blocks, D-Mannitol, GOD, and Fe3O4@PEI nanoparticles (Figure 12A). This gel produced heat by being placed under an alternating magnetic field by existing nanoparticles, which, in addition to the hyperthermia effect, led to a gel-sol phase transition and penetration into tumor cell spaces (Figure 12B), which is due to the presence of D-Mannitol, which causes Dehydration of the cell and its contraction could be intensified. Then, glucose and O_2_ in the components and GOD together produced H_2_O_2_ during reaction $glucose+O_{2}+H_{2}O\to gluconic\;acid+H_{2}O_{2}$ . Then nanoparticles in this acidic environment accelerate the production of ·OH according to the Fenton $2H_{2}O_{2}\to {HO}^{.}+{HOO}^{.}+H_{2}O$ reaction, which increases the oxidative stress of the tumor and damages the heat shock protein 70, which is highly expressed in hyperthermia. Hyperthermia itself increases the activity of nanoenzymes (Wu et al., 2019).

Wang et al. (2020a) developed a gel based on catechol functionalized chitosan and MnO2 nanosheets for treating skin cancer and multidrug-resistant (MDR) bacteria wound healing, which could self-assemble, and in addition, the nanosheets created an oxidative cross-link in it. This redox-responsive gel reduces the hypoxia in tumor areas with a high concentration of H2O2 by converting it to O2. In addition, increasing the level of O2 improves the effect of the DOX drug contained in the gel. On the other hand, nanosheets with a photothermal effect with good temporal and spatial control cause hyperthermia in the tumor, which can almost completely remove the tumor. In addition, due to the presence of positively charged chitosan and nano-knife-like MnO2 nanosheet, this gel can remove bacteria long-term by contacting them. In addition, with the destruction of the gel and due to the enzymatic effect of nanoplates, the consumption of ROS reduces inflammation and accelerates the proliferation of fibroblasts, and as a result, wound healing improves.

As an alternative to conventional surgical closure or implant fixation methods (sutures, staples, meshes, and grafts), Zhou and colleagues prepared surgical sealants based on ECM-derived biopolymers gelatin and chondroitin sulfate, which have a Schiff-base dynamic bond under shear and adhesive properties. This gel can reattach torn tissues, close bleeding wounds, leak fluids, and laparoscopic surgery sealant in elastic tissues. This gel had good biocompatibility, biodegradability, and elasticity. Besides, it was economical and easy to synthesize and use. The Schiff-Base bond and hydrogen bond between gel and tissue at body temperature could be weakened by lowering the temperature to 20°C and causing gel separation. The concentration of the gel and the degree of oxidation of chondroitin sulfate could adjust the hydrogel’s mechanical properties and adhesion strength (Zhou et al., 2021). Table 2 presents a summary of the characteristics of the gels formed by the action of different stimuli.

**TABLE 2:** A summary of the characteristics of the gels formed by the action of different stimuli.

Type of stimuli	Gelation mechanism	Advantage	Disadvantage	Gelation speed	Most used polymers
temperature	LCST: Reduction of polymer-water interaction and increase of polymer-polymer hydrophobic interaction with increasing temperature	Control of drug release with volume changes	The possibility of toxicity and non-degradability of the gel and blocking the syringe during injection	high	LCST: PNIPAM
	Thermoreversible: micellization due to polymer-polymer interaction and then establishing a link between micelles				Thermoreversible: pluronic
pH	Removal of positive covalent charges and formation of hydrogen bonds between amine groups with increasing pH	The possibility of using to treat cancer and inflammation due to the special ph of these diseases	The possibility of inflammation due to the acidic pH of the sol	less than the temperature stimulus	chitosan
ion	Establishing an ionic bond between the anion resulting from the dissolution of polymers and some special cations		Low cell adhesion	less than the temperature stimulus	Alginate, pectin
			Low degradation rate of alginate		
enzyme	Accelerating the creation of bonds between biomolecules by enzymes	Biocompatibility			Some protein and polysaccharide polymers
		Mild temperature and ph conditions			
		Low toxicity			
photo	The transformation of the primary substance into a free radical and its propagation	Controlling the time and place of radiation	The possibility of damage to the cargo due to the increase in temperature caused by the high gelation speed, damage to the DNA of the cell due to the presence of UV light and free radicals, and the limitation of use due to the low penetration depth of UV light	Too high	Some acrylated polymers
Shear stress	Viscosity increase and heal with the end of injection shear stress	Long life of gel due to self-healing, the low effect of injection shear stress on cells		good	

## Summary, challenges and future perspective

Smart injectable gels are polymer networks containing a fluid that can be injected into the body in a solution state and then turned into a gel due to the effect of a stimulus *in situ*. The most important advantage of these materials is the delivery of therapeutic agents in a process with minimal invasiveness. The process of gelation of these materials can be under changing conditions inside the body, that is, with hydrophobic interactions during temperature changes, ionization of polymer side chains with pH changes, creation of ionic bonds with changes in the concentration of polyvalent ions, Or the application of external stimuli, i.e., light radiation, reduction of shear stress and increase of gel viscosity, generation of heat in the temperature-responsive gel by nanoparticles under magnetic field, or biological stimulation of transglutaminase enzymes can cause gel formation. Each method has its characteristics, advantages, and disadvantages, which this article discusses.

Despite the many advantages of these materials in medical applications, their properties can be improved by considering some things. First of all, like other biomaterials, in the design of gels, we must pay attention to the compatibility of the gel with sensitive cells to remain healthy and maintain their proliferation, as well as compatibility with sensitive molecules such as DNA, oligonucleotides, proteins, and peptides to maintain activity them and not to denature them. Also, degradation products, remaining components, and catalysts or cross-linkers that have not reacted should be investigated in terms of causing cytotoxicity. Also, the interactions between the material and the host tissue should be considered to prevent inflammation. To help the proteolytic degradation of the gel, the remodeling phenomenon, and the penetration of cells due to the signals secreted from the cell, it is possible to use peptides sensitive to matrix metalloproteinase in the gel, inspired by the biological phenomena. In addition, regarding degradable materials, it should be noted that the rate of degradation in the drug delivery application should be adjusted according to the rate of release of the drug in our consideration and the application of tissue engineering according to the rate of formation of new tissue.

Rheology is the following important parameter in the development of gels. If the viscosity of the material is lower than a certain limit, the gelation speed decreases. On the other hand, gels with high viscosity require more force for injection, which is unpleasant for both the patient and the operator. Also, using this method for deep target locations that require long transportation of materials in the catheter is impossible. In this context, using a two-syringe tool shortens the transfer distance, but there is a possibility of accidental changes in viscoelasticity or phase separation due to the reaction or interaction of the mixing components in this case. The rheology of these materials must be carefully adjusted so that the material flow is homogeneous. Mechanical properties are one of the other challenging areas, and to strengthen this property in gels, stronger gels, which probably have more compact networks, can be used. However, in this case, it is harder to penetrate the interface of the extruded flows, and the possibility of creating a homogeneous network is less. On the other hand, the desired gel must have good flexibility to remain intact after reorganizing the infiltrating cells.

The phase transition behavior of a copolymer system may change upon the addition of drugs, cells, or even a cell culture medium. In addition, the release of cells and GFs, and drugs at the desired place and time is still a remarkable field. Among the shortcomings of the hydrogels made so far, we can mention their short half-life. Also, delivery to the target location will not be suitable for multiple injections. In addition, in natural polymers, there is a possibility of changes between different batches and, as a result, properties with a change of origin. Increasing the concentration of GF for human use is another challenge.

Developing these materials leads to producing new materials with complex formulations that have safety risks and low repeatability of properties. Therefore, it is always necessary to use simple formulations along with simple manufacturing methods. Despite this issue, hybrid hydrogels, composite systems with nano or microparticles containing drugs or proteins, materials responsive to multiple stimuli, and hydrogels with the ability to identify biomolecules have unique properties that can be used to solve many challenges have been mentioned.

Recent publications on hydrogel research reveal complex polymers with several parts. In the envisioned application, each component has a specific function. A hydrogel, for example, having diverse properties (e.g., hydrophilic and hydrophobic monomers) exhibits distinct surface qualities. Another polymer can be grafted onto the surface of a hydrogel to alter its surface characteristics. Another method to change surface properties is to physically alter the gel surface. The order of the monomer units in a hydrogel can also be altered, as can the cross-linking process. The mechanical properties of a hydrogel are directly affected by cross-linking. These alterations can have an impact on the hydrogel’s physical qualities as well as its biodegradability. Combinatorial synthesis can be employed because it might be challenging to find the ideal monomer sequence or the right combination of cross-links to produce the required properties. In combinatorial synthesis, a wide range of monomers, modifiers, cross-linkers, and other components can be used. The majority of current research, as shown by recently published studies, integrates various modification procedures to produce a single hydrogel with as many desired properties as possible. Due to compatibility issues, however, it is not straightforward or simple to combine different approaches. The absence of commercially viable hydrogel products necessitates new research efforts and the continuation of existing hydrogel programs.
